# "The Hospital" Nursing Mirror

**Published:** 1897-07-10

**Authors:** 


### "The Hospital" Nursing Mirror.

Being the Nursing Section of "The Hospital."

[Contributions for this Section of "The Hospital" should be addressed to the Editor, The Hospital, 28 & 29, Southampton Street, Strand, London, W.C., and should have the word "Nursing" plainly written in left-hand top corner of the envelope.]

### News from the Nursing World.

### SOLDIERS AND SAILORS WHO FOUGHT FOR THE QUEEN.

On July 5th the Prince of Wales inspected 400 soldiers and sailors, veterans, representatives of the battles of the reign, at Chelsea. The Princess received purses for the Homes for Nurses of the Soldiers and Sailors' Families' Association, and the garden fête was made bright by entertainments given by well-known artistes.

### MATINEE IN AID OF THE QUEEN'S NURSES.

A number of artistes gave their services on June 28th at a matinée at the Tivoli Theatre of Varieties. Mr. Vernon Dowsett was the manager, and the directors of the Tivoli gave the use of the theatre. The programme was long and popular, and the performance altogether a success. The attendance, including that of the Duke and Duchess of Teck and the Duchess of Connaught, was excellent, in spite of raised prices. The decorations were beautiful, the boxes being festooned with freshly-cut roses.

### THE JUBILEE FETE AT SWANSEA.

On June 22nd and 23rd a most successful fête was held in the Victoria Park, Swansea. In spite of the oppressive weather on the second day, the attendance was large, and there was a handsome sum to be handed over to the hospital for the benefit of which it was organised.

### LADIES AT THE MOSCOW MEDICAL CONGRESS.

Amongst the arrangements made for the International Medical Congress in Moscow, in August, the ladies have not been forgotten. A ladies' committee has been formed, under the presidency of the wife of the president of the Organising Committee, and under the patronage of the Grand Duchess Elizabeth. An exhibition, illustrative of Russian women's work, is to be held, and a handbook of interesting information is to be distributed amongst the lady guests.

### SUCCESS AT GLASTONBURY.

The dwellers in the neighbourhood of Glastonbury have adopted the scheme to establish a nursing home to celebrate the record reign. A cottage is to be either built or bought, and the services of a fully-qualified nurse provided. It is intended to provide three beds for the reception of patients. £350 was estimated to be necessary to establish the hospital—a remarkably small sum for the purpose—and £85 has been collected in annual subscriptions. The institution is to be called the Victoria Nursing Home, and a committee of 26 ladies is to be organised, charged with the responsibility of providing delicacies for the patients in succession. We can only hope, for the sake of the patients and the general harmony of the arrangements, that very definite lines will be drawn up upon which the provisions shall be supplied.

### THE LOSS OF THE "ADEN."

Another of those terrible disasters which come so unexpectedly and appal the stoutest hearts has been placed to the record of losses at sea. It is five years since the Peninsular and Oriental Company lost the *Bokhara*, and now the *Aden*, homeward bound from Yokohama, has fallen upon an evil fate on the rocks that gird the eastern side of the island shore of Socotra. Amongst the numerous victims we see the name of Mrs. Smyth, wife of Dr. Smyth, medical missionary at Ningpo, with those of several ladies of the Church Missionary Society. So far nothing has been heard of the boat which managed to put off, and as the *Aden* struck the reef at three a.m. on June 9th the worst possible fears prevail.

### THE HAMPSTEAD REJOICINGS.

Dr. W. Heath Strange has presented to Hampstead Hospital a valuable picture in commemoration of the year. On Jubilee Day each patient was allowed two guests to tea. Before they left, Bishop Wakefield's hymn and the National Anthem were sung in the wards.

### ARMAGH'S NURSES.

Miss Dunn, superintendent of Queen's Nurses in Dublin, pleaded very successfully for the institution of a branch of the Jubilee Nurses' Institute at Armagh. She received a hearty welcome, subscriptions for the work flowed in, and two nurses will soon begin their work in a district that wants them badly.

### NURSES' INSTITUTE AT WEDNESBURY.

The scheme of celebrating the Queen's reign by erecting a nurses' institute has been warmly taken up by the inhabitants of Wednesbury. The foundation-stone was laid on June 22nd amidst loyal and enthusiastic demonstrations. A procession was formed at the Town Hall by the Mayor, Councillor Oldbury, corporation, volunteers, fire brigade, postmen, and others, which passed through cheering crowds to the site of the new building in Walsall Road. Here the Mayor gave an address, performed the ceremony, and was presented by the hon. architect, Councillor Joynson, with a trowel and mallet.

### A CHILDREN'S SALE OF WORK.

The children of Scarborough have a hospital league which appears popular. With a view to planting practical sympathy for the poor in the hearts of the little ones, the Rev. W. Francis organised a system of regular almsgiving; and this year, in honour of our Queen, each child member of the league prepared some piece of work within its capacity, the result being the Jubilee sale of work held in the central hall, the out-patients' waiting-room, and the nurses' dining-room of the hospital, on June 26th. The Mayoress performed the opening ceremony. We are glad to observe that indiscriminate begging by the children is discountenanced, and that the league is regarded as a means of inculcating true charity in the characters of its members.

### THE WORKING LADIES' GUILD.

THE summer sale of the above guild was held on June 30th and July 1st and 2nd at 1, Belgrave Square, S.W. The work improves in quality, and that shown was quite up to the best professional standard. The prices, too, were most moderate. Some old needlework had been repaired by members of the guild with great success. The headquarters are at 251, Brompton Road, which are well worth a visit.

### PARSEE PHILANTHROPY.

A PARSEE lady, Bai Motlibai, whose recent death deprives Bombay of an active philanthropist, recognised the value of the devoted work of the nurses who fought the plague, and expressed a keen desire to show her appreciation of their services in a practical form. Her son, Mr. N. M. Wadia, C.T.E., has written to the Plague Committee asking in what manner he can best fulfil his mother's wish, and General Gatacre, whilst deeming the question one for the public to answer, suggests that anything that would give Bombay a supply of well-trained nurses would confer a boon upon the city; he also points out the need of a nurses' sanitorium on the hills.

### RETURN OF THE NURSES FROM GREECE.

THE nurses who went to the front during the war are now for the most part homeward bound, if they have not already arrived. The wounded soldiers are either well, or drafted into the ordinary military hospitals, or have passed over the "narrow stream," and their English attendants, having accomplished their work of mercy, return to "the common round the daily task," let us hope, with that humility born of wider experience which despises not the thing of apparently small importance.

### THE RELIGIOUS DIFFICULTY ONCE MORE.

ENDEAVOURS are being made to institute a trained district nurse at Armagh. A correspondent of the *Armagh Standard* takes umbrage at one of the rules of the Nurse Training Society, which forbids the nurse "pointing the patient to the patient's Almighty Physician," maintaining that "it requires a little grain of 'real mustard seed,' such as some of my brethren in the T. C. ministry do not value, to be sown at each sick-bed." It is well to avoid reefs, which in stormy weather wreck the ship, and we would point out that it is a nurse's business to nurse the sick, and a minister's work to point out to the patient the Almighty Physician; that however desirable it may be and is from every point of view that a nurse should possess true religious faith, yet we maintain that to avoid "reefs," it is necessary that she should refrain from teaching and preaching, and content herself with being that most powerful argument for religion—a good example; and that she should permit her patients to enjoy unmolested the ministrations of the clergy of their own creed.

### FREE NURSES FOR HUDDERSFIELD.

THE chairman of the Free Nurses' Committee, Mr. Chas. E. Freeman, announces that a sum of £2,500 has been collected for the fund. He points out that this sum, at the present low rate of interest obtainable, is not enough to support a sufficient number of nurses to attend all the sick poor of the town, and he therefore appeals for more liberal support.

### A NURSING INSTITUTION FOR CHESTERFIELD.

THE foundation of the "Victoria Home for Nurses" is occupying the attention of Chesterfield. The objects of the new home are to provide a home where reliable nurses can be obtained on an emergency, to secure the nurses a comfortable residence, and to supply and support a district nurse; £250 has been obtained, with the promise of another £100 if a like sum be subscribed. The scheme seems sure of success, and the sanguine-minded already see the germ of a cottage hospital in the new institution.

### FEVER-STRICKEN INNISKEA.

INNISKEA is an island off the coast of Ireland, the inhabitants of which are in a state of wretched poverty. They are living in hovels without windows, and barely high enough to stand up in. Fever has broken out amongst them, and forty-eight persons are attacked. The City of Dublin Nursing Institute has sent two nurses to the place, where they arrived, after a long, rough journey, on Saturday, June 12th.

### WHY HAS ADEN NO NURSES?

ATTENTION has been drawn to the fact that when the survivors of the *Aden*, after 19 days' exposure, landed at the port of the same name there were no nurses to look after them. This is the only disaster marring the Jubilee rejoicings, and attention is called to the efforts made by those who live "on the barren rocks of Aden" to obtain help in this particular. There are two hospitals at Aden, the Civil Hospital, with 98 beds, under the medical direction of Surgeon-Captain J. L. T. Jones; and the European General Hospital, with 26 beds, of which Surgeon-Major T. Cummin, V.C., and Philip Dias, assistant-surgeon, constitute the medical staff. When we remember that in some illnesses nursing is more than half the battle, there is no need to expatiate of the crying want of good nurses. Nevertheless, the climate is one of the worst, the intense heat is most exhausting, so that white nurses might not be able to fulfil the arduous duties of their profession under such conditions Surely, though, it might be possible to maintain a staff of trained native women, under the direction of a European matron.

### THE NEEDS OF THE PRISON INFIRMARIES.

THE question of nursing prisoners is one beset with many difficulties, but the country men and women of Mrs. Elizabeth Fry ought not to rest until they are satisfactorily overcome. The prison medical authorities would welcome trained assistance, but the trained nurses must be prepared to undertake the duties of warders as well. Women may as well make up their minds, however, that even as they have invaded the medical profession, so men are taking up work in the nursing world, and in the male wards of the prison infirmary only men will be admitted.

### SHORT ITEMS.

THE authorities of the Dewsbury and District Infirmary mark the year by arranging to give the nurses a week's extra holiday; the domestics will also be allowed a longer spell of freedom.—Nurses Fletcher and Black, sisters at Prince Alfred Hospital, Sydney, left for England on the "Darmstadt," on the 22nd ult., in order to take a special course of midwifery nursing at one of the large home hospitals.

### Practical Aspects of a Nurse's Life.

By Sister Grace.

### II.—PROBATIONERS IN THEIR FIRST YEAR.—*Continued.*

### Bedmaking.

The probationer's work begins at seven a.m. with bed-making, and in this you will be helped by a more experienced probationer, or by the staff nurse. You will soon learn to perform this duty quickly and well. I think three-quarters of an hour is about a fair time to allow for making the beds of thirty patients—that allows an average of one minute and a half to each bed; some do not take so long. Some are already made by the patients who have vacated them, but others will take as much as five minutes, when the occupants are feeble and slow in their movements. Never permit yourself to show impatience with the deliberation of the sick. Many can only have their draw sheets pulled through or changed, and the top clothes turned and smoothed, and many, especially in the surgical wards, cannot be moved at all except by very skilled hands, and are left until the sister comes to attend to them herself. On entering the ward strip all the beds that are vacant, so that they may have a brief airing, and then go steadily round, making all the beds in rotation. You will have instruction at the time from those with whom you work; listen carefully to what they tell you, so that you do not cause discomfort to the patients. When they are allowed to get out of bed see that they are warmly wrapped up in a blanket, and that their bare feet do not rest on the floor. Be also very careful that there is no exposure of the helpless patients; it is quite unnecessary, and betokens great clumsiness and want of that delicacy of feeling that should distinguish every nurse.

### Washing and Preparing Patients.

This is a much more onerous affair in the women's ward than with the men. Their heads of long hair require constant and patient attention, and, when ill, the smallest pull or jerk of the head is a great trial to the sufferer; they are frequently fussy and exacting, but now is the time to begin to exercise the virtues of patience and gentleness. Very many patients require not only the ordinary washing, but a very thorough cleansing from head to foot and an entire change of clothes from the nature of their illness. The moving of helpless patients at first presents many difficulties to the beginner, but you will be taught, and practice makes perfect.

In most hospitals the wards are ready for the house surgeons and physicians by 9.30 or 10 a.m. During their visits to the wards you will probably have to fetch and carry screens, but otherwise will not at first come in contact with the doctors. A senior probationer or the staff nurse assists the sister in waiting upon them. After all the dressings have been done, and before the visiting staff arrive, the ward must be lightly swept again, to get rid of the atoms of cotton wool and flue that have accumulated under the beds.

### Patients' Dinner and Tidying of Ward.

About midday comes dinner, which is served by the sister. Pay particular attention to what she says as to the destination of each plate, so that she may not have to speak twice on the same subject. After this you will assist in a general tidying of the ward and in attending to the various requirements of the patients. Then will come your own dinner, for which half an hour is allowed, after which you return to the wards. You will see the sister or staff nurse give the medicines; notice how careful they are not to administer any dose without looking at the label, however well they may know the case and the dose. Accidents may very readily arise from neglecting this precaution.

### Time Off Duty and Evening Work.

Some time during the afternoon you will probably have two hours off duty. Try to spend this time as much as possible in the open air. When you are tired with hard work, to lie down is a great temptation, but resist it and go out and fill your lungs with oxygen, and thus you will return to your ward lively and fresh instead of jaded and weary.

You will soon learn to prepare the patients' tea, and to cut the bread and butter dexterously. Study their different tastes as to thick and thin; a little thought and kind attention to these trifles, and others of a like nature, make the difference between a popular and unpopular nurse.

The evening work greatly resembles the morning, and both morning and evening work, generally speaking, is somewhat heavier in the surgical than in the medical wards on account of the frequently recurring dressings, but the greater fatigue is balanced by the interest which centres round more active work.

### Amount of Study Desirable.

You will find all the backs are rubbed carefully to avoid bed-sores. This is always done twice a day, and in the more helpless cases three or four times. Bed-sores are the terror of all nurses; I shall speak to you more on this subject later on. Once a week, oftener if possible, the patients' feet are washed; this needs a light and firm hand; it is very unpleasant to have a nurse with a hesitating and uncertain hand.

The patients are all settled for the night by eight o'clock, and usually about three times a week every probationer is free at that hour; one of these evenings should be spent in study. I do not think any great amount of reading is beneficial to a probationer in her first year's training; she does not see enough of the cases to derive much benefit from reading about them, but at the end of the twelvemonth she begins to see more; her ward duties become all easy routine, and she is able to assimilate the knowledge she gains from books.

Do not undervalue this period of early training; you should be gaining in self-control, and the habit of unquestioning obedience, and gaining accurate knowledge of how unimportant you are personally, and yet how greatly the comfort of the patients depends upon your conscientious discharge of duties that bring you no praise or recognition. You are like the workman who greases the wheels of the locomotives; few give a thought to him, and yet if he neglected his duties he would cause fatal accidents.

### Minor Appointments.

Essex and Colchester Hospital.—Miss Mary Alabaster was appointed Charge Nurse of the Essex and Colchester Hospital on July 5th. She was trained at the Royal United Hospital, Bath, where, with her three years' certificate, she was awarded the silver medal given by the president of the hospital for general proficiency.

Nurse Ida Mathers has been appointed Staff Nurse at the Hospital and Home for Incurable Children, 2, Maida Vale, W. Miss Mathers was trained at Ayr County Hospital.

Miss Sarah Richmond, superintendent night nurse City of London Union Infirmary, has been promoted to the position of Assistant Matron.

### Wants and Workers.

[The attention of correspondents is directed to the fact that "Helps in Sickness and to Health" (Scientific Press, 28 & 29, Southampton Street, Strand, London, W.C.) will enable them promptly to find the most suitable accommodation for difficult or special cases.]

Has any kind lady or gentleman a bed rest they could dispense with for the use of a very poor helpless young woman of 29, who could enjo a little change of position if she could be propped up against a rest.

### Post-Graduate Clinics for Nurses.

By a Trained Nurse.

### XXI.—VARIETIES OF REST-CURE.

One of the most depressing features of rest-cure nursing is the fact that, however great the benefit obtained, unquestionably serious relapses do frequently take place, and many of our best cases were patients who "took the treatment" regularly every two years, or even more frequently than this. And they were perfectly aware when discharged as "cured" that they were in reality only effectually patched, and realised that in all probability they would gradually relapse into their original state in about two years—or even sooner than this if any strain or extra anxiety were put upon them.

The normal history among the women revealed that the majority of cases broke down under some long-continued worry or anxiety. Sometimes these took the form of a large sorrow, such as the death of husband or child. But much more commonly the mind had been for some length of time harassed by an accumulation of small frets and trivial worries. To some temperaments the larger tragedies of life are much easier to bear than continually arising daily worries. And there is no question that to do one's daily work, while at the same time "something is on the mind," has a terribly deteriorating effect upon the constitution. Working with a worried mind has been compared to that condition into which a machine gets when it is running with dry and dusty bearings. Now, when all the oil is worn from the bearings these grind together and wear one another out. Similarly it is the extra expenditure involved in striving against difficulties and making efforts when the mind and vitality are depressed which does the mischief to the individual. Now, in family life, there are frequently jars and irritations which have the same effect as happens to the bearings of bicycles—to use a familiar instance—when these are allowed to run without oil. And because of such frictions and worries it becomes absolutely necessary to remove a patient needing rest-cure in the full significance of the remedy from their normal and every-day surroundings. Most of us have experienced the terribly depressant effect which some particular room or set of surroundings may have on the temperament, just because these surroundings have an association which is unhappy or irritating. And we know what marvels are wrought on health and happiness by change of air and scene, just as we are familiar with the fact that many a sick person who is cured of the acute symptoms of his disease shows no real evidence of convalescence till he is removed from his sick room, with all its wearisome associations and recollections of sleepless, weary nights and hours of aches and unrelieved pain and discomfort.

Dr. Weir Mitchell is very emphatic as to the hopelessness in nearly every instance of treating a rest-cure patient amid his home surroundings. And a very favourite axiom of his is that the average rest patient from his trying temper and worrying ways has a baleful effect on the health of a household. He says, "where there is one hysterical girl, sooner or later there will be two sick women."

Dr. Weir Mitchell in his treatment of rest-cases invariably directs the attention of the nurse in charge as much to the importance of mental as to physical rest. And the nurse will find in the majority of cases that the mind of her patient will cause infinitely more trouble and anxiety than will the physical state. And in order that the mind may have that freedom from care which is so important a factor of the system, seclusion and removal from home is the first essential stage in the proceedings of rest-cure. I have many times seen the attempt made to treat rest patients in their normal surroundings. In these cases their pleadings to "be allowed to stay at home" had been sufficiently pathetic to overcome their doctor's experienced conviction of the difficulties in the way of a rest-case treated at home. If the patient be a woman she promises readily and faithfully not to worry. But such a promise is easier made than kept. For even in a normal state of health it is sometimes difficult enough not to worry. But with the nerves in the exaggerated condition of sensibility characteristic of these cases, the patient strives in vain to "keep quiet and not bother." Every knock at her front door, every unexplained noise rouses her to interest. She is firmly impressed with a conviction that husband and children are being starved or neglected, and nothing will shake her belief that the whole household is going to wreck and ruin just because she is forbidden to take any part in its management. In her own house, too, she is a person of authority. She can send for servants, cross-question them on domestic questions, and issue instructions on culinary or household matters. And, though every care be taken, it is impossible that the baby's every cry can be kept from her. The least incident of that kind is enough to set her overwrought nerves to thrill, and produce so much excitement as to undo the good resulting from the skilful care both of the doctor and nurse. And since a great many of the cases we are considering are the victims of hysteria, the very first salutary step is taken when measures are directed towards the prevention of that picturesque posing so dear to the heart of the hysterical patient, whether man or woman. The abnormal craving for sympathy so marked in persons suffering from nervous exhaustion is another obstacle to be overcome, and this is well-nigh impossible so long as the patient remains at home. In addition to these very important considerations comes the question of disciplining and managing the individual—that mental and moral influence which is so hard to maintain, but which is so absolutely essential to the real success of the case. This rule and discipline which, under the Weir-Mitchell treatment, the nurse has so rigorously to keep, may often seem needlessly severe to injudicious and morbidly sympathetic relatives who fail through ignorance of the science involved in the treatment to grasp the necessity of such hard and fast ruling. Surrounded by affectionate relatives the rest-cure patient will give way to emotional and passionate outbreaks, and will invariably appeal from the judgment of doctor and nurse to the clemency of friends. Oliver Wendell Holmes, in his graphic and picturesque way, has described some types of rest patients as "Vampires who suck the healthy blood of those around them." And in a sense this really takes place. I have repeatedly seen a whole household broken down in health and spirits through a kindly attempt on the part of the doctor to accede to the wish of a nervous patient to be "rest-cured" at home. I have never seen a real success resulting. Either a complete breakdown of the attempt takes place after two or three weeks and the patient is removed to a rest-cure home to complete recovery, or, if the patient be allowed to remain at home during the whole six or eight weeks of treatment, the cure will probably be only partial and the patient merely "patched up." In many of these cases of part cure some discredit is naturally brought on the principle of rest-treatment, when in reality the failure has resulted only from an attempt to carry it out under conditions which do not conduce to success.

### THE NEW HOSPITAL FOR WOMEN.

The percentage of mortality at the New Hospital for Women is stated to be very low; out of ninety important operations there have been only two deaths.

### The Power of Management.

By a Matron.

Nursing may be styled an art, but the management of a ward becomes a science. There are many who may practise the art successfully, who will fail to treat it scientifically. Those destined to be pioneers and leaders are those who can make their profession a science—that is to say, who are able to reduce their knowledge and skill to a system. Private nurses should be artists, ward sisters should be scientists. Each requires a different standard of mental ability, and to a certain extent of physical fitness. Some are born to lead, others to obey.

Every nurse must acquire a thorough practical knowledge of her duties, but those who are to fill the higher appointments must also possess great judgment and special powers of management. A sister's work consists of far more than the mere nursing of the patients, or the apprehension of the technicalities of her profession; she must be able to conduct the affairs of her ward systematically and to direct and control all working under her; she must have the art at her fingers' ends, but she must also be able to impart the science. She must feel the responsibility of wielding aright the immense power entrusted to her. By her judicious management of work, nurses' and patients, the wheels run smoothly, friction is avoided, happiness promoted, and the commonweal preserved.

The chief difficulties lie with the people, not with the things. True the ward itself requires management, it must be cleaned and tidied, and kept in order and repair. Its work must be regulated by hours and events, and it must always be ready for inspection; but what are details such as these compared with the task of governing men and women of all classes, ages, and temperaments? To begin with, men must never suspect they are being managed, or they at once become intractable. How often will a house surgeon or physician find one of his wards easy to work in, though strictly disciplined, whilst another is always difficult, though the laws are those of the Medes and Persians? In the one case the sister studies his wishes and understands his method of working; in the other, she is tactless and injudicious, enforcing the letter rather than the spirit of the law. Students are generally outspoken critics, and do not hesitate to express their views on ward administration. They are entitled to consideration, and readily appreciate the wards where facilities are offered for the accomplishment of a large amount of work in a short space of time. The staff nurses need direction and control, but a tactful sister will hold the reins firmly, but not too tightly. She will let them work with her, albeit under her supervision. She will seek to gain both their confidence and their affection. The probationers require a tighter hold, a more vigilant eye to prevent catastrophes born of ignorance. Encouragement with them works better than praise, but it is well to lead rather than to drive. In nothing does the management declare itself more forcibly than in the behaviour of the patients. It is no light matter to induce a number of sick people to live contentedly by rule and *régime*, to teach them to control themselves for the sake of others, to keep them happy, satisfied, brave, and unselfish; to make the children quiet, the women cheerful, and the men disciplined; yet it may all be done by the influence of one tactful woman.

There are many who can steer a ship in a calm sea who would let it sink in a storm; there are others who will only wake up when a difficulty arises, and are content to drift purposeless when the wind is fair. Good management is needed for plain sailing, and in an emergency it is essential. Examples might be cited *ad lib.* of the catastrophes which have happened, of the predicaments that have arisen, involving increased labour and anxiety, of tempers lost and hasty words spoken, all from lack of good management and want of tact. The corresponding triumphs of those who rule with judgment and keep themselves in touch with all are not so obvious, simply because everything works so smoothly and systematically there is no occasion for comment. Nevertheless appreciation is not lacking, though it may be felt rather than spoken.

It is this power of management which signalises the career of a successful ward sister, and makes all who work with her and under her understand how great things may be accomplished quietly, without agitation or fuss.

### Queen Victoria's Jubilee Institute for Nurses.

ROLL OF QUEEN'S NURSES, July 1st, 1897.

Her Majesty has been graciously pleased to approve the appointment of the following as "Queen's" Nurses, to date July 1st, 1897:—

England. — Superintendent: Catherine G. Evans, Gloucester. Nurses: Jane Taylor, Euphemia Wills, Constance M. Thompson, Florence Frost, Alice M. M. Tilbury, Eveline M. Pemberton, Florence Andrees, and Annie E. Colville, London; Ethel Sharpe, Barnet; Mary Goffe, North Finchley; Florence G. Beeston, Kingston-on-Thames; Mabel Hawkes, Buntingford; Mary E. Clemitson, Dunmow; Emily Cutt and Gertrude Dunn, Stamford; Florence W. Pritchard, Rushden; Lilian M. Morgan and Ethel F. M. Blair, Chatham; Emma Dudley, Brighton; Alice N. Pipe, Southampton; Edith H. Pearse, Penryn; Florence L. Salmon, Leighton Buzzard; Grace Smith, Sleaford; Mary Burton, Edensor; Catharine W. Lyon, Hull; Fanny E. Whitfield, Warrington; Lydia O. Dixon, Bacup; Mary G. Peel, Bolton; Blanche Hendy and Alice L. Gibbons, Stoke-on-Trent; Annie M. Peters and Clare L. Bradshaw, Hanley; Jane M. Tilburn and Evelyn I. Pocock, Bury; Queenie Midlaine and Alice M. Carlton, Torquay; Amy Tandy, Birmingham; Clara H. Jones, Mary de A. Willett, and Mary McKay Wight, Liverpool; Sabina Moore, Martha Suggitt, and Elizabeth B. Hill, Manchester; Winifred Rogers, Rosalie F. L. Palk, Minnie Wells, and Sara J. Courteen, Gloucester.

Wales.—Nurses: Margaret J. Pugh, Sarah A. Read, and Mary J. Hughes, Cardiff; Sarah E. Bottany, Bangor; Katharine Sykes, Panteg.

Scotland.—Nurses: Agnes S. Eadie and Caroline Maxwell, Edinburgh; Margaret H. Watt, Susan K. Lindsay, Agnes Ramsay, and Agnes Binney, Glasgow; Harriet Menmuir, Anna Rodney Hunter, and Frances C. Crouchley, Dundee; Mary Berretti, Aberdeen; Elisabeth McCalman, Perth; Christina Jackson, Blairgowrie; Eliza J. Kenmuir, Larbert; Margaret D. Easton, Carriden; Barbara L. Clark, Alyth; Jean Robinson, Airdrie; Jane H. Mills, Lanark; Annie M. Nicolson, Iona and Bunnessan; Marie L. Nicolet, Dysart; Bertha Röber, Coltness; Margaret McIntosh, Beith.

Ireland.—Nurses: Anna G. Laidlaw, Kathleen M. M. Martin, and Josephine de S. Purcell, Dublin; Mary Gilbert, Ballycastle; Kathleen G. Browne, Limerick.

### A THOUGHTFUL INVITATION.

Messrs. Hitchcock and Williams, whose stands at the recent Jubilee celebrations commanded one of the best views of the ceremony before St. Paul's, kindly invited some 2,000 nurses from various metropolitan hospitals to witness the rehearsal of the great procession. The act was rendered more kindly by their providing refreshments for their guests.

### Everybody's Opinion.

[Correspondence on all subjects is invited, but we cannot in any way be responsible for the opinions expressed by our correspondents. No communication can be entertained if the name and address of the correspondent is not given, or unless one side of the paper only is written on.]

### THE TRAINING OF MALE NURSES.

"A THANKFUL MALE NURSE" writes: I feel extremely gratified by your article, entitled "A Plea for Male Nurses," and I know I speak on behalf of a great number of my colleagues, who, like myself, would have been only too pleased, after training in the institution you mention, to have entered a general hospital. The question is, Has the attempt to train and establish male nurses in English general hospitals been tried? If so, under what auspices, and with what result? Of course, one knows that a number of objections can always be raised where men and women work together; but in the cause of nursing the sick I am confident in most instances that male and female nurses working side by side would become a boon, both to the patient and the nursing profession in general. Nursing naturally is a woman's work, but in certain cases where a great deal of tact and firmness is required (not mental cases) I have invariably found that they are apt to forget their managing capabilities, and therein have been pleased to act in subjection to their fellow male nurses. As matters are at present, we, as male nurses, are hardly entitled to the name, except to nurse such cases that came under our notice during our probation in a special hospital. I sincerely trust that you may see your way clear to publishing this letter in your valuable paper, and I hope it may be the means of establishing a new feature and a necessary want in the nursing world, and that it may be found that men can nurse, and would, therefore, be a great help to noble women who spend their lives in the cause of the sick and suffering. Some would say that men could not be trained for nurses because they are not steady; of course that could be very easily guided over by making a stipulation that they must be temperate, then I think there would be no question as to their not being generally recognised in the nursing profession.

### NARROW-MINDED NURSES.

"ONLOOKER" writes: As a new reader of your paper I have read with great interest and some amusement the correspondence on "Narrow-minded Nurses" which has been going on. May I take this opportunity for asking wherein lies their narrow-mindedness? Is it in a nurse's natural inclination when thrown with others of her profession to "talk shop" and discuss the events passing round her in the vocation she has chosen? Surely not. No one would think of blaming or bemoaning when they hear the clergyman talk enthusiastically about his services, clubs, and guilds, or the sporting man about his hunting, his fishing, and polo; no one would denounce the society girl as "narrow-minded" for expatiating on her last ball or the delights of Ascot or Henley; or the 'Varsity undergraduate for waxing hot over his boating and cricket, or his prospects in his "schools." They are simply displaying a natural interest in what to them makes up that medley of complex circumstances which is "life." Surely the hospital nurse is equally right in her enthusiasm for her profession—an enthusiasm which must obviously lead her into living simply and solely in the little hospital world around her and into taking, unintentionally perhaps, but a meagre interest in the burning question, the *sturm und drang* of the larger world outside its walls. What follows? The hospital nurse goes home for three weeks' holiday. She finds things discussed of which she has heard nothing or, perhaps, only by an accidental rumour. Events in the political, social, or religious world, the new novel, the last plays at the theatre, the freshest vagaries of fashion or art . . . she feels strangely ignorant and strongly resembling the proverbial fish out of water. Those outside, and perhaps inside, her family find her a trifle dull, the "nursing craze" has left her terribly uninteresting and with "absolutely nothing to say." And is the nurse to blame? Surely not. The busy, active life, with its few minutes daily for reading up for the next examination—the daily round of duties which must be done—a monotonous routine, indeed, to those who have not found it gilded by love to God and man; others must, of necessity, find those engaged in it in more or less of a groove. It seems to me such a necessity that it is one of the things which the would-be probationer must take into account when considering the pros and cons of the life before her. It is an impossibility to live two lives—one inside and one outside the hospital walls—but at the same time there is no need to ignore the fact that there are matters of vital interest going on in the outside world in which interest the hospital nurse may share, though to be absolutely in touch with them and up-to-date is out of the question. And is there much to be missed in so doing? Has the nursing profession so few compensations as to allow of the smallest shade of regret at the loss of a certain up-to-date smartness in conversation, &c.? I am sure there are hundreds of nurses, even with the smallest experience of hospital life, who would gladly answer in the affirmative, and even glory in their so-called "narrow-mindedness."

### Appointments.

### MATRONS.

NORTH STAFFORDSHIRE INFIRMARY AND EYE HOSPITAL, STOKE-ON-TRENT.—Miss S. A. Warburton was appointed Superintendent of Nurses at the North Staffordshire Infirmary and Eye Hospital, Hartshill, on July 1st. She was trained at the London Hospital, and has successively held appointments as charge nurse and night superintendent, City of Dublin Hospital; lady superintendent, Dublin Orthopædic Hospital; matron, R. M. Hospital, Stepney Causeway, E.; and matron of City of London Infirmary, Bow Road, E.

MONSALL FEVER HOSPITAL, MANCHESTER.—Miss E. M. Roberts was appointed Lady Superintendent of Nurses at the Monsall Fever Hospital on June 30th. She was trained at the Nightingale Home, St. Thomas's Hospital, and afterwards held the positions of theatre sister and sister of the medical and surgical wards of the hospital.

HOSPITAL FOR INSANE, RYDALMERE, ON THE PARAMATTA.—Miss Lena Pope, one of the sisters of the Prince Alfred Hospital, has been appointed Matron. This lady was trained under Miss McGahey, and adds another to the list of good appointments obtained by nurses trained at the above-named hospital.

EASTERN FEVER HOSPITAL, HOMERTON, N.E.—Miss Flora Masson has been appointed Matron of the Eastern Fever Hospital. She was trained at St. Thomas's Hospital and the Nightingale School, and has been until her present appointment matron of Radcliffe Infirmary, Oxford.

THE COTTAGE HOSPITAL, HINCKLEY.—Miss Y. M. Johnson was appointed on July 2nd Matron of the Cottage Hospital. She was trained at Birmingham General Hospital, and has been charge nurse at Eston Hospital.

### Presentations.

MR. T. JACKSON, senior surgeon, on behalf of the employés of the London and North-Western Railway who have been patients at the Wolverhampton General Hospital, presented Nurse Annie McLaren with an illuminated address. A large and sympathetic assembly were present, as the services of Miss McLaren in the men's accident ward for many years have been greatly appreciated by all with whom she has had to do. She took up her work in 1873, and since has devoted her energies to nursing the most severe cases admitted into the hospital. She has seen the advent of six matrons, and the growth of the institution from 70 to 230 beds.

EDINBURGH.—Last month the members of the medical and nursing profession connected with the Edinburgh City Hospital presented their senior staff nurse, Nurse Gilchrist, with a beautiful silver tea service—tea kettle, teaspoons, and a toast rack—on her appointment as matron of the Portobello Fever Hospital. Bailie Pollard (chairman of the committee) made the presentation, which was accompanied by an illuminated address signed by nearly ninety members of the staff (past and present). Nurse Gilchrist has worked for over 18 years in the City Hospital, and often under great difficulties. She will be much missed on the staff. She carries with her the best wishes of her fellow workers, the committee and medical superintendent, and matron, who were very glad to be able to show in a small way their respect and affection for her.

### Greek Ladies as Red Cross Nurses.

By an English Nurse at Athens.

At the commencement of the war between Greece and Turkey a Greek Women's Union was formed, consisting of considerably over one hundred Greek ladies, many of them occupying high positions in society, and under the immediate superintendence of the Princess Sophia. This union was formed to take charge of the nursing and care of the sick and wounded. They organised hospitals in places where necessary, and the amount of work done by them and the way it was done is worthy of the highest admiration. One or two English nurses who were at the time in the East, and who had had considerable experience in nursing abroad, joined the movement and worked with the Greek ladies from first to last. As early as April 8th, Mrs. Ralli, Mrs. Palli, Miss Eliopoulos, Dr. Mary Kalapouthaki, Miss Palmer, and Miss Rider left Athens on the Royal yacht *Sphacteria* for Volo, where a house had been lent to Princess Sophia for the purpose of establishing a Red Cross hospital. Volo was chosen because the military authorities had refused to have nurses sent to the frontier. On arriving at Volo we found the house so beautifully painted that it seemed a great pity to use it for a hospital, but failing to find a more suitable building we were obliged to avail ourselves of it. By April 13th we had everything quite ready and our first three patients arrived, sent down by Dr. Galvani, the director of the Red Cross Society. We had a very busy week, beating mattresses, shaking blankets, getting rooms ready, arranging and organising for the inauguration of the hospital and also for the nurses coming from Athens. Princess Sophia then arrived with five nurses, Miss Bull, a voluntary nurse, and a Greek voluntary nurse, just in time for the inauguration. Simultaneously with their arrival came seven wounded insurgents. We kept two of the English nurses, and Sister Francis Dunbar from Syra also stayed. The remaining three English nurses and Miss Bull proceeded to Larissa the next day to take charge of the military hospital there.

The wounded came in so fast to Volo that we had to telegraph for some of the Greek ladies and some Greek nurses to come from the Civil Hospital at Athens and help us.

On the 16th four more nurses left England and arrived at Volo about the 23rd, and were put to work in the military hospital, which was close to our Red Cross Hospital, and consequently they messed with us. Miss Boyd, an American lady, who had been studying archæology in Athens, also came to Volo and worked most pluckily and entirely alone in another large house which had been taken for a military hospital. The worst cases were brought to the Red Cross Hospital, as it was the only one with an operation-room, and the patients were shipped on to Athens as soon as possible to make room for new-comers. As the trains arrived from Larissa containing the wounded, we from the Greek Ladies' Union met them at the station with ambulances and medical comforts. As there was not a carriage to be had in Volo, all having been sent up to Larissa, there was a procession pitiable to see of the halt and maimed (using in some cases their rifles as crutches) upon the arrival of the trains each evening.

There were eight hospitals in Volo in addition to tents erected, and the majority of these hospitals were under the military authorities, nursed by orderlies, and consequently in a deplorable condition.

A week after the departure of the nurses for Larissa early one morning, to our utter amazement, we saw a regular stampede of carriages, carts, horses, mules, &c., laden with people and luggage, all making towards Volo as fast as they could go, and presently a train arrived with the Red Cross doctors and nurses, who brought all their patients from Larissa down to Volo. The medical cases were put into a field adjoining the Red Cross Hospital, where they were obliged to wait till the tents were fixed up.

The Consul at Volo advised the English nurses to leave at once for Athens, as it was thought to be unsafe to remain longer in Volo. Consequently all the English nurses from Larissa and those from the military hospital left in the hospital ship which was lying in the harbour. So great was the panic in Volo that the ship was filled to overflowing, people climbing up the sides, and it was impossible to get all the wounded on board, and most had to return to their different hospitals. Thus only the staff of the hospital of the Greek Ladies' Union was left to look after *all* the remaining sick and wounded, and Miss Boyd, who still pluckily stuck to her post, since she had been unable to ship off any of her patients. Besides our own work, we had very little time to do more than just go through the tents each day and see that the patients had food and drink, as most of our servants had left us and the work at this time was frightfully heavy. What was most astonishing to the onlooker was that these Greek ladies, who ran so much more risk than the English nurses, remained at their posts and behaved splendidly during this most trying time, doing excellent work. It was three days after this before any vessel came to relieve us of more of our patients, and, as can be imagined, the strain was terrific, especially as we were expecting the Turks down on us every moment. Too much cannot be said in praise of the English correspondents, who promised faithfully that when the Turks arrived they would come straight up to the Red Cross Hospital and do what they could for us and our sick. For the next fortnight we were receiving wounded from Pharsala and Velestino, where the Greeks had retreated from Larissa, and these we also had to keep until the hospital ships turned up from Athens to take them down to the hospitals there, and so make room for fresh arrivals. Volo suffered from many panics during this time, and we were often advised even by telegram from Athens to pack up and return, but of course with the work we had on our hands retreat was impossible, and, as it turned out, unnecessary at that time, and it was only the day before the Turks entered Volo that we shipped our last patients, and the staff of the Ladies' Union and Miss Boyd left with stores the same day for Athens.

The four English ladies who had remained during this time volunteered to stay longer in case any more wounded turned up from Velestino, under charge of Mr. Noel (a gentleman who had joined the Red Cross Society), and also to save the military stores if possible, as we had not forgotten how, in the retreat from Larissa, the most valuable ambulance stores, including 150 stretchers, instruments, lint, bandages, drugs, &c., fell into the hands of the Turks, and the next day was spent in collecting the most valuable of the stores from the different military hospitals, and the one or two English gentlemen resmaining in Volo lent valuable aid. In less troublous times much amusement might have been caused by this procession of Englishmen and nurses carrying tents, poles, and hospital furniture to a paddock adjoining the English Consulate, where a guard was put over them. The next day a caique was obtained at an exorbitant price to take them to Stylitha, where the military doctors now were stationed. All this time we were watching the steady advance of the Turks coming down the hill beyond the town, and the moment the captain of the caique discovered their near approach he took alarm and fled, leaving the rest of our stores on the shore, so we had to gather them up and return them to the care of the Consul and his guard. We might, perhaps, have got into the caique and sailed away too, but we had promised the few remaining peasants in Volo, who were in a state of terror at the approach of the Turks, that we would remain behind.

As the Turks arrived we set out to meet them. They were quite orderly and very anxious to show that they intended to do no harm to the inhabitants, and were generally on their best behaviour. We were three days in Volo with them and they paid us every attention. The Red Crescent (the Turkish Ambulance) called on us and invited us to help them nurse the 200 wounded they were bringing down from Larissa to our vacated hospitals. However, we did not feel inclined to do this, as our sympathies were with the Greeks, and we were naturally anxious to find some means of rejoining them. On the third day to our delight we were able to hire a small caique, which had returned with the first peasant family who had summoned up courage enough to venture back to their home in Volo. After spending 24 hours in this open boat we arrived at Stylitha, glad enough to be once more among the Greeks. Dr. Bellini, the head military doctor there, arranged for us to go to Domoko should fighting recommence, but the Crown Prince was against our doing so; consequently, we had to be content to work in the military hospital in Lamia during that last disastrous battle of Domoko. The miserable state in which the poor soldiers were brought in is indescribable; some of them were already delirious, and many had been without food for two or even three days. Of course all thought the Turks might be in the town any minute, hence it was imperative to get the patients out of the place as quickly as possible. Mrs. Cassaretti and her band of workers, all Greek except Miss Boyd, who had joined her after leaving Volo, had a very hard time as their hospital was full to overflowing, and when the order came to close it their patients, including many bad cases, had to be moved under great difficulties down to the port, where several ships were waiting to take the wounded, some to the *Daily Chronicle* Hospital at Chalcis, under the charge of Mr. Abbott, but the majority to Athens, where every comfort awaited them, not only in the military hospitals, where the majority of the English nurses were working, but in the many large buildings fitted up as temporary hospitals by the Red Cross Society, where the patients were devotedly nursed by the Greek ladies themselves, who left children and homes in order to help their poor countrymen, and really to work day and night whenever the necessity arose. It is to be regretted that more has not been recorded about the admirable work done by Greek ladies during the recent war, and one is struck by the accounts in the English papers, from which one is led to suppose that all the work was done by English nurses, though there could not have been more than thirty-five of them altogether. When one thinks of all that these Greek women went through, all that they did, and how splendidly they behaved under the most trying circumstances, too much cannot be said in their praise. English nurses being sent out by an organised fund could not possibly have felt all that this war meant as the Greek women, whose hearts were being torn by seeing their beautiful country at the mercy of the Turk, and knowing the many dangers to which their husbands and brothers were being exposed. Many of these ladies were hurt that the English nurses did not more fully grasp the painful position in which Greece and the whole Greek nation were placed. When nurses are chosen for these expeditions they should be chosen for their high character, tact, and good breeding, in addition to their nursing qualities, and they should try to fully enter into the feelings of the people they are working for. Never should they forget that the English nurse abroad is an object of criticism as well as interest, and if she wishes to uphold the nobility and dignity of her profession, and win the love and esteem of her foreign sisters, she must make her conduct such as to command respect, and her work her first consideration; and give up any pleasures for the time being, if necessary—be ready to give up her life itself should duty demand.

### The Medical Work of the Zenana Mission.

THE annual report of the Zenana Bible and Medical Mission, or Indian Female Normal School and Instruction Society, for the year ending December 31st, 1896, is issued, and the chronicle of the Medical Mission contains much of interest. At Lucknow a new wing was opened in November in connection with the Lady Kinnaird Memorial Hospital. It contains two wards of eight beds, three of two beds, an isolation ward of four beds, and the necessary baths and offices. This brings the accommodation up to fifty beds. The cost was nearly £1,000, of which about £300 is still to be subscribed. Miss Sheldon is in charge of the nurses, and reports great improvement in the work done by native nurses. Miss M'Dowell, M.D., who was at Benares Hospital and Dispensaries, has been obliged by reason of ill-health to pay a long visit to America. She is back at work again, and Miss Pailthorpe, M.B.Lond., who undertook her work remains to help her. There have been many changes on the staff at the Duchess of Teck Hospital, Patna. Miss Mackinnon, L.R.C.P. and S., and Miss Gregory both left in the spring on furlough, and Miss Gray and Miss Ferguson took charge. Both ladies hold the L.R.C.P. and S. diplomas.

### Royal British Nurses' Association.

THE annual meeting of members of the Royal British Medical Association will be held at the East Conference Hall, Imperial Institute, South Kensington, on Thursday, July 22nd, 1897, at eleven a.m. Agenda: 1. To receive the annual, financial, and other reports. 2. To elect the general council for the ensuing year. 3. And such other business which may be advertised in the medical papers. The luncheon will be served in the same building at half-past one p.m. Tickets to admit members and friends, price 2s. each, may be obtained of the secretary, 17, Old Cavendish Street, W. By kind permission of the authorities of the Imperial Institute, members of the R.B.N.A. can obtain admission to the exhibition, after the luncheon, at the reduced rate of 6d. each. The hon. treasurer and Mrs. Langton will be "At Home" to members of the association at 62, Harley Street, Cavendish Square, W., at which reception H.R.H. the President has graciously signified her intention of being present. Tea, four to seven p.m.

### Where to Go.

THE Educational Department of the Earl's Court Exhibition, under the presiding genius of Lady Warwick, has arranged an "Educational Congress on Subjects of Imperial Interest" on July 12th, 13th, and 14th. The admission to visitors of the exhibition is free. At 2.30 on July 13th Mrs. Henry Fawcett will take the chair; and the subjects discussed will be "Medical Training for Women in England," after a paper by Mrs. Garrett Anderson, M.D.; "Medical Women in India," after one by Mrs. Scharlieb, M.D.; and "The Care of the Sight in Childhood and Youth," after one by Miss Ellaby, M.D.

